# Enhanced external counterpulsation improves dysfunction of forearm muscle caused by radial artery occlusion

**DOI:** 10.3389/fcvm.2023.1115494

**Published:** 2023-03-02

**Authors:** Zhenyu Wang, Chun Yao, Lihan Huang, Jianwen Liang, Xiaocong Zhang, Jian Shi, Wenbin Wei, Jing Zhou, Yahui Zhang, Guifu Wu

**Affiliations:** ^1^Department of Cardiology, The Eighth Affiliated Hospital of Sun Yat-sen University, Shenzhen, Guangdong, China; ^2^Department of Cardiology, Foshan Fosun Chancheng Hospital, Foshan, Guangdong, China; ^3^Department of Cardiology, Affiliated Hospital of Yan’an University, Yan’an, Shaanxi, China; ^4^School of Rehabilitation Sciences and Engineering, University of Health and Rehabilitation Sciences, Shandong, China; ^5^Guangdong Innovative Engineering and Technology Research Center for Assisted Circulation, Sun Yat-sen University, Shenzhen, Guangdong, China; ^6^NHC Key Laboratory of Assisted Circulation, Sun Yat-sen University, Guangzhou, Guangdong, China

**Keywords:** enhanced external counterpulsation, radial artery occlusion, oscillatory shear, pulsatile shear, hemodynamic, human umbilical vein endothelial cells, shear stress

## Abstract

**Objective:**

This study aimed to investigate the therapeutic effect of enhanced external counterpulsation (EECP) on radial artery occlusion (RAO) through the oscillatory shear (OS) and pulsatile shear (PS) models of human umbilical vein endothelial cells (HUVECs) and RAO dog models.

**Methods:**

We used high-throughput sequencing data GSE92506 in GEO database to conduct time-series analysis of functional molecules on OS intervened HUVECs, and then compared the different molecules and their functions between PS and OS. Additionally, we studied the effect of EECP on the radial artery hemodynamics in Labrador dogs through multi-channel physiological monitor. Finally, we studied the therapeutic effect of EECP on RAO at the histological level through Hematoxylin–Eosin staining, Masson staining, ATPase staining and immunofluorescence in nine Labrador dogs.

**Results:**

With the extension of OS intervention, the cell cycle decreased, blood vessel endothelial cell proliferation and angiogenesis responses of HUVECs were down-regulated. By contrast, the inflammation and oxidative stress responses and the related pathways of anaerobic metabolism of HUVECs were up-regulated. Additionally, we found that compared with OS, PS can significantly up-regulate muscle synthesis, angiogenesis, and NO production related molecules. Meanwhile, PS can significantly down-regulate inflammation and oxidative stress related molecules. The invasive arterial pressure monitoring showed that 30Kpa EECP treatment could significantly increase the radial artery peak pressure (*p* = 0.030, 95%CI, 7.236–82.524). Masson staining showed that RAO significantly increased muscle interstitial fibrosis (*p* = 0.002, 95%CI, 0.748–2.128), and EECP treatment can reduce this change (*p* = 0.011, 95%CI, −1.676 to −0.296). ATPase staining showed that RAO significantly increased the area of type II muscle fibers (*p* = 0.004, 95%CI, 7.181–25.326), and EECP treatment could reduce this change (*p* = 0.001, 95%CI, −29.213 to −11.069). In addition, immunofluorescence showed that EECP increased angiogenesis in muscle tissue (*p* = 0.035, 95%CI, 0.024–0.528).

**Conclusion:**

EECP improves interstitial fibrosis and hypoxia, and increases angiogenesis of muscle tissue around radial artery induced by RAO.

## Introduction

In 1989, Campeau reported for the first time that it was conducted coronary angiography through transradial artery access (TRA) (1). The advantages of no braking after TRA operation contribute to improved patient comfort, reduced bleeding and hematoma, shortened hospital stay and reduced medical costs (2). In addition, TRA also reduced mortality in high-risk patient subgroups, such as those presenting with acute coronary syndromes (3, 4). In 2018, the European Society of Cardiology and the European Association of Cardiothoracic Surgery recommended TRA as a preferred approach for coronary artery diagnosis and treatment (5).

Radial artery occlusion (RAO) is the most frequent post-procedural complication of TRA. Several studies have demonstrated significant structural changes after TRA catheterization (6, 7). The primary mechanism of early RAO after TRA consists of acute arterial thrombosis, resulting from the combined effect of catheter-related endothelial and vessel injury, local hypercoagulable state, and decreased blood flow from compressive hemostasis (8). Most early RAO patients are missed due to radial artery palpation. Chronic RAO will account for a large proportion of these patients. It can be caused by the proliferation of vascular smooth muscle and the progressive thickening of the intima media caused by proliferation (6, 9). Several studies have shown that the expression of important active factors such as nitric oxide (NO) and vascular endothelial growth factor (VEGF) in vascular endothelial cells is decreased (10, 11), and the expression of inflammatory and procoagulant molecules nuclear factor-kappaB (NF-κB), Von Willebrand factor (vWF) and tissue factor (TF) is increased (8, 12, 13), which may be involved in the occurrence of RAO. Relative studies shows that RAO can be prevented effectively by adequate procedural anticoagulation (14, 15), proper transradial artery duration and magnitude of compression (16, 17), and reduction of sheath and catheter size (6, 18). However, the increase in the number of percutaneous coronary intervention and non-standard operations still result in a large number of RAO patients. Although asymptomatic from an ischemia standpoint in the vast majority of cases, it precludes ipsilateral TRA for future procedures. If there is symptomatic hand ischemia, it may be necessary to reopen an occluded radial artery for transradial procedure (19, 20). This will undoubtedly increase the related vascular complications again.

Oscillatory shear stress (OS) will be generated at the stenotic artery, which will lead to vascular dysfunction and atherosclerosis, and may further aggravate the degree of arterial stenosis (21). Conversely, physiologically high shear stress is protective, which can improve vascular endothelial function and reduce atherosclerosis. Among them, endothelial cells are critical sensors of shear stress (22). Enhanced external counterpulsation (EECP)is a non-invasive pneumatic technology. It can not only effectively increase diastolic blood pressure, mean coronary artery pressure and coronary artery flow, but also reduce main artery systolic blood pressure by controlling a series of lower limb cuffs to inflate in the diastole and deflate in the systole, generate characteristic double pulse blood flow. Our previous animal studies had demonstrated that EECP intervention could inhibit intimal hyperplasia, restore vascular endothelial function by increasing pulsatile shear (PS) stress and modifying shear stress responsive gene expression, attenuate atherosclerosis progression through modulation of proinflammatory pathway, as well as promote coronary collaterals and angiogenesis (13, 23–25).

As far as we know, there is no noninvasive treatment that can significantly improve RAO. In this study, we analyzed the transcriptome of human umbilical vein endothelial cells (HUVECs) from the longitudinal time series and horizontal comparison through the OS and PS model *in vitro*. Furthermore, we further studied the evidence that EECP can improve RAO at invasive hemodynamic and histological levels through animal experiments.

## Materials and methods

### High throughput sequencing data archives

The expression profiles by an array of GSE92506 were retrieved from GEO database. To profile shear stress-regulated endothelial transcriptomes, researchers performed RNA-seq with HUVECs subjected to different shear flow conditions, including atheroprotective PS (12 ± 4 dyn/cm^2^) and atheroprone OS (0.5 ± 4 dyn/cm^2^), or kept as static control for four time periods (1, 4, 12 and 24 h). The Platform of high throughput sequencing is GPL15433 (Illumina HiSeq 1000, Homo sapiens). Series matrix files and data table header descriptions of GSE92506 were downloaded from the GEO database to describe the change trend of differential molecules through transcriptome time-series analysis of OS intervention HUVECs. In addition, we have compared the differential molecules between PS and OS at 24 h.

### Data transformations and differential molecular screening

We used the R package “DEseq2” (v3.6.3) to analyze the differentially expressed genes (DEG) between OS and ST at different time points (26), and found that the genes with p.adj < 0.05 and Log2 (Flod Change) > 1 or < −1 had significant differences. R package “Mfuzzy” (v2.20.0) was used to cluster the standardized high-throughput sequencing data of DEG (OS vs. Static) with fuzzy C-means to describe their changing trend over time. The minimum centroid distance of a series of clusters was calculated and classified into different clusters. In addition, we also analyzed the differences between OS and ST at 24 h, DEG was presented by volcano map.

### Pathway enrichment and dynamic analysis

We used Gene Ontology/Kyoto Encyclopedia of Genes and Genomes (GO/KEGG) (27). The pathway with p.adj < 0.05 is considered to be significantly enriched. The direction of the pathway was calculated using the average value of the difference multiple of the important molecules (p.adj < 0.05) in the significantly enriched pathway. Compared with baseline, all significant mRNA were used as the background for pathway dynamic analysis (28).

### Animals and groups

A total of 9 female Labrador dogs (24 weeks old, average weight 19 ± 0.4 kg) were purchased from Jinan Jinfeng Experimental Animal Co., Ltd. and raised in Shenzhen Leading Medical Service Co., Ltd. The rearing environment: temperature (22–28°C), humidity (50–70%), density (2 dogs/cage), free drinking water, feeding (2 times/day), lighting time (07:30–19:30). The animals were randomly divided into three groups (each group *n* = 3): Control, RAO and RAO-EECP. The animal experiment part of this study has been approved by the Medical Research Ethics Committee of the Eighth Affiliated Hospital of Sun Yat-sen University (Futian, Shenzhen) (Research Ethics of the Eighth Affiliated Hospital of Sun Yat-sen University 2021-037-01).

### Radial artery hemodynamic measurements

Hemodynamic parameters of the left radial artery during EECP intervention were measured in 3 dogs to determine the best counterpulsation pressure. Intramuscular injection of 0.5–1 mg/kg (Jilin Fanggong, China) of xylazine hydrochloride injection was used for induction anesthesia, followed by endotracheal intubation for ventilation (Mindray veta5, China). After that, isoflurane (Baxter Healthcare, United States) was used for continuous anesthesia to maintain SO_2_ at 100%. The animal was then placed in supine position on the counter pulsation bed, and a set of modified dog-specific cuff was wrapped to closely fit the lower limbs and buttocks of the dog for EECP (PSK P-ECP/TM Oxygen Saturation Monitor, China). The cuff was inflated with compressed air from the distal end to the proximal end successively in early diastole, and deflated rapidly before systole of the next cardiac cycle. The invasive arterial pressure of the left radial artery was monitored with a multi-channel physiological monitor (BIOPAC, MP150) to evaluate the appropriate counterpulsation pressure for EECP treatment.

### RAO model and EECP treatment

The left radial artery of 6 dogs was ligated, and the RAO-EECP group was performed 30Kpa EECP for 60 min every day within 14 days. In order to eliminate possible circadian influence, EECP were conducted at the same time of every day.

### Tissue preparation

All dogs were anesthetized with xylazine hydrochloride injection (Jilin Fanggong, China), at the same time, all animals were euthanized by intravenous injection of excessive 10% potassium chloride at the same time as well. At the end of the experiment, the lateral muscle samples of left forearm of all dogs were taken immediately and washed in cold normal saline. The sample of each dog was divided into four parts, which were fixed with 4% paraformaldehyde and then embedded in paraffin for HE staining and collagen staining, embedded with OCT embedding agent (SAKURA, Japan) and frozen in liquid nitrogen for ATPase staining (29). In addition, another 4% paraformaldehyde samples were used for immunofluorescence.

### Morphological evaluation

The detailed experimental protocol of Hematoxylin–Eosin staining, Masson staining, ATPase staining and immunofluorescence are showed in the Supplemental material.

### Statistical analysis

We conducted Shapiro Wilk normality test for all quantitative data. For normal distribution samples, Student’s *t*-tests and ANOVA were used to compare parameter data between two conditions and multiple conditions, respectively. The homogeneity of variance was evaluated by Levene’s test. For ANOVA analysis, multiple hypothesis test (Tukey HSD test) was used and corrected by Bonferroni method. In addition, we used the R package “ggplot2” (v3.3.3) and GraphPad Prism (v9.1.1) to visualize the above statistical analysis data.

## Results

### Time-series mRNA expression and pathway dynamic enrichment analysis

**Figure 1:**
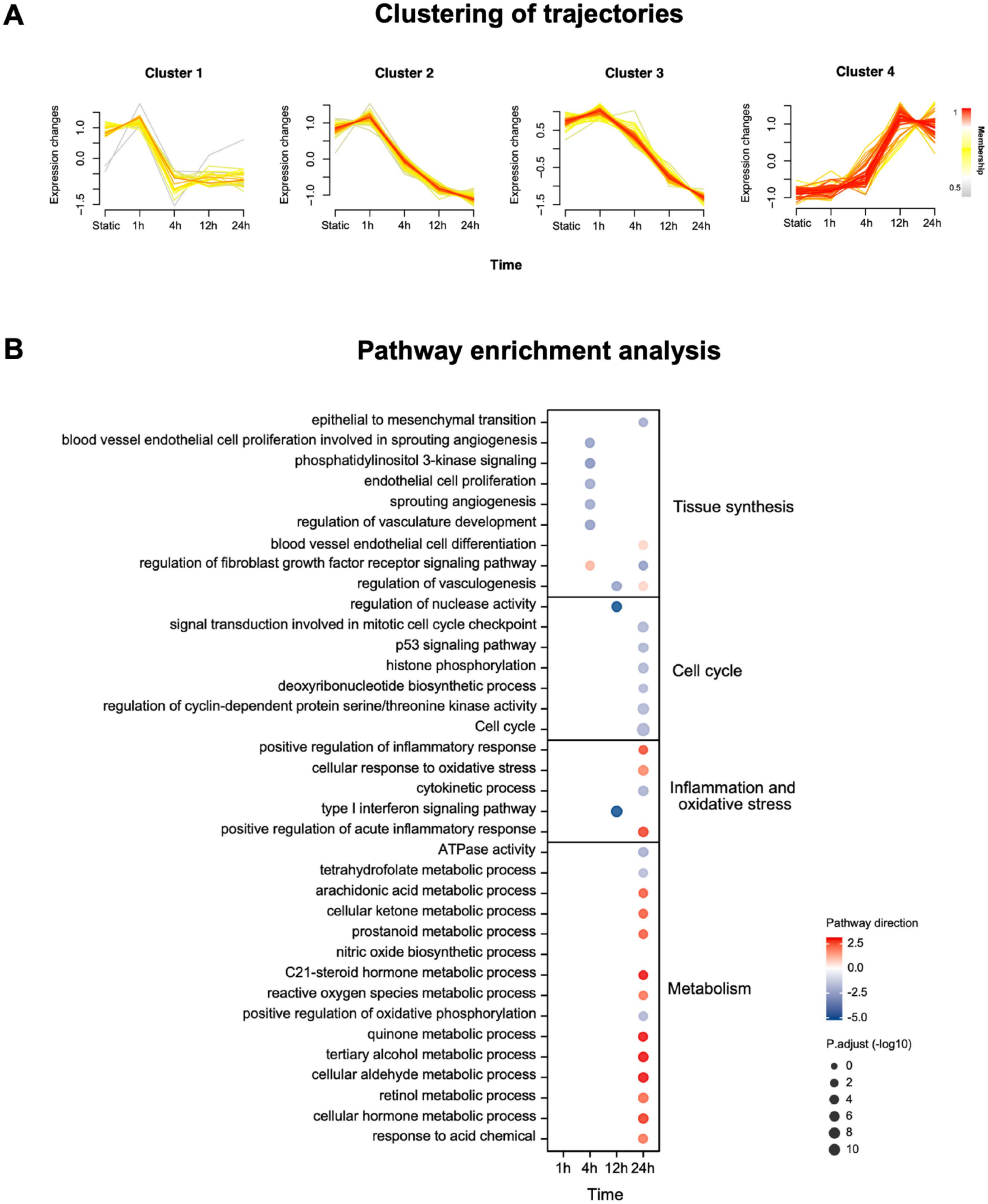
mRNA expression and enrichment of HUVECs under OS intervention. **(A)** Clustering of longitudinal gene expression trajectories (p.adj < 0.05). Membership represents the association strength between the sample and the cluster. **(B)** Pathway enrichment analysis using mRNA significantly changing in tissue synthesis, cell cycle, inflammation and oxidative stress and metabolism (p.adj < 0.05, |Log2 Fold change|>1). Pathway direction is the mean log2 fold change relative to baseline of significant transcripts in each pathway (blue, downregulated; red, upregulated). The dot size represents pathway significance.

The important molecules (p.adj < 0.05) at each time point compared with static under OS intervention were divided into four main longitudinal trajectories by C-means clustering (Figure 1A) according to their expression trend. We can find that the expression of molecules in Cluster 1, 2 and 3 reached the peak at 1 h, and then the expression of molecules in Cluster 1 decreased rapidly at 4 h, and then slowly recovered, while the expression of molecules in Cluster 2 and 3 decreased slowly after 1 h until 24 h. On the contrary, the molecular expression in Cluster 4 was gradually up-regulated until it reached peak at 12 h.

At each time point, we use all important mRNA (p.adj < 0.05) to conduct enrichment analysis on dynamic pathways related to tissue synthesis, cell cycle, inflation and oxidative stress and metabolism (Figure 1B). The results showed that with the extension of OS intervention, the cell cycle decreased, blood vessel endothelial cell proliferation and angiogenesis responses were down-regulated. By contrast, the inflammation and oxidative stress responses and the related pathways of anaerobic metabolism of HUVECs were up-regulated. The details of time-series mRNA expression and pathway dynamic enrichment analysis were in Supplementary data sheet.

### Horizontal comparative analysis

**Figure 2:**
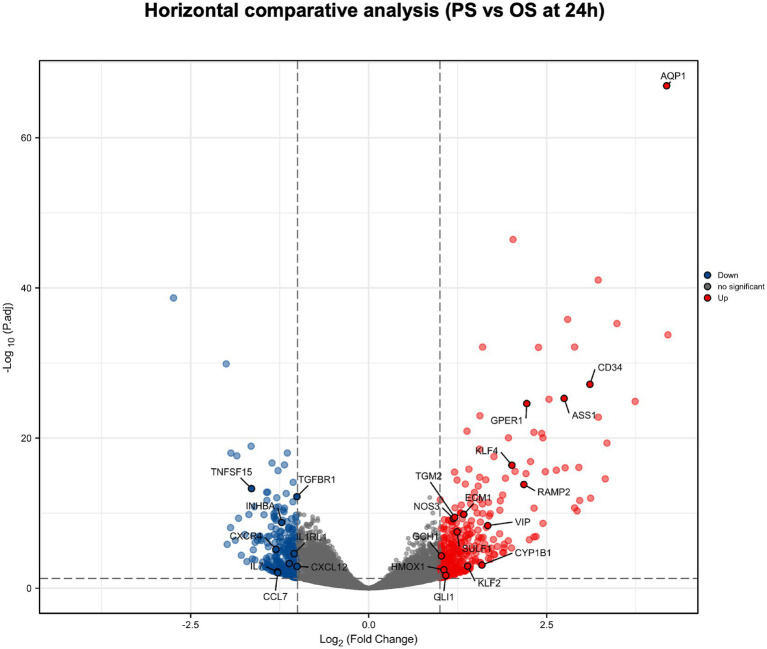
Horizontal comparative analysis of PS and OS in HUVECs mRNA. Volcano plots show the significant differences between PS and OS in HUVECs mRNA at 24 h. Each dot represents a mRNA (blue, downregulated; red, upregulated; gray, no significant). In HUVECs mRNA, the molecules with significant difference were defined as p.adj < 0.05 and |Log2 Fold change|>1.

We performed a differential analysis on mRNA of HUVECs after 24 h PS and OS intervention (Figure 2). We found that compared with OS, PS can significantly up-regulate muscle synthesis related molecules (such as *GLI1*, *TGM2*, *SULF1*, etc.) (30–35), angiogenesis related molecules (such as *CD34*, *CYP1B1*, *AQP1*, *ECM1*, *GPER1*, *HMOX1*, *RAMP2*, etc.) (36–48), and NO production related molecules (such as *ASS1*, *GCH1*, *NOS3*, *KLF4*, *KLF2*, etc.) (49–56). In addition, PS can significantly down-regulate inflammation and oxidative stress related molecules (such as *IL7*, *INHBA*, *CCL7*, *CXCL12*, *TGFBR1*, *CXCR4*, *IL1RL1*, *TNFSF15*, *ITGA4*, etc.) (57–72).

### Radial artery hemodynamic measurements

**Figure 3:**
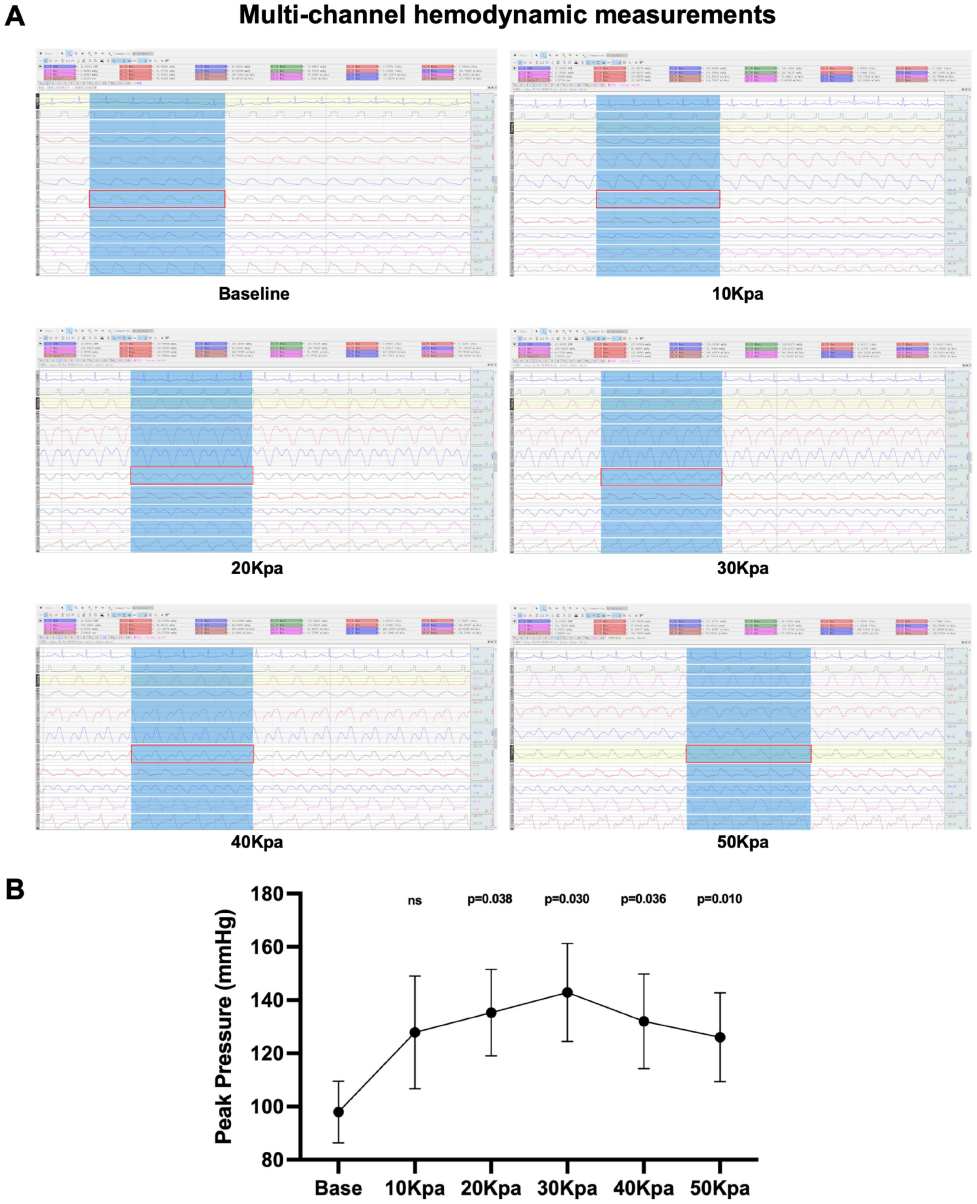
Multi-channel hydrodynamic measurements under EECP intervention. **(A)** Multi-channel hydrodynamic measurements of different counterpulsation pressures. The eighth channel (red rectangle) is the radial artery pressure. **(B)** Peak pressure of radial artery under different counterpulsation pressure. Data were expressed as means ± SD (*n* = 3). Results were evaluated by Student’s *t*-test (each counterpulsation pressure with baseline).

Multi-channel physiological monitor shows the arterial flow and pressure in multiple parts of the body, including invasive radial artery pressure, under the intervention of different counterpulsation pressures (Figure 3A). We found that EECP can produce characteristic double pulse blood flow, and the radial artery peak pressure is the highest under the intervention of 30Kpa counterpulsation pressure (*p* = 0.030, 95%CI, 7.236 to 82.524). We took 30Kpa as the subsequent counterpulsation pressure parameter (Figure 3B).

### Morphological evaluation

**Figure 4:**
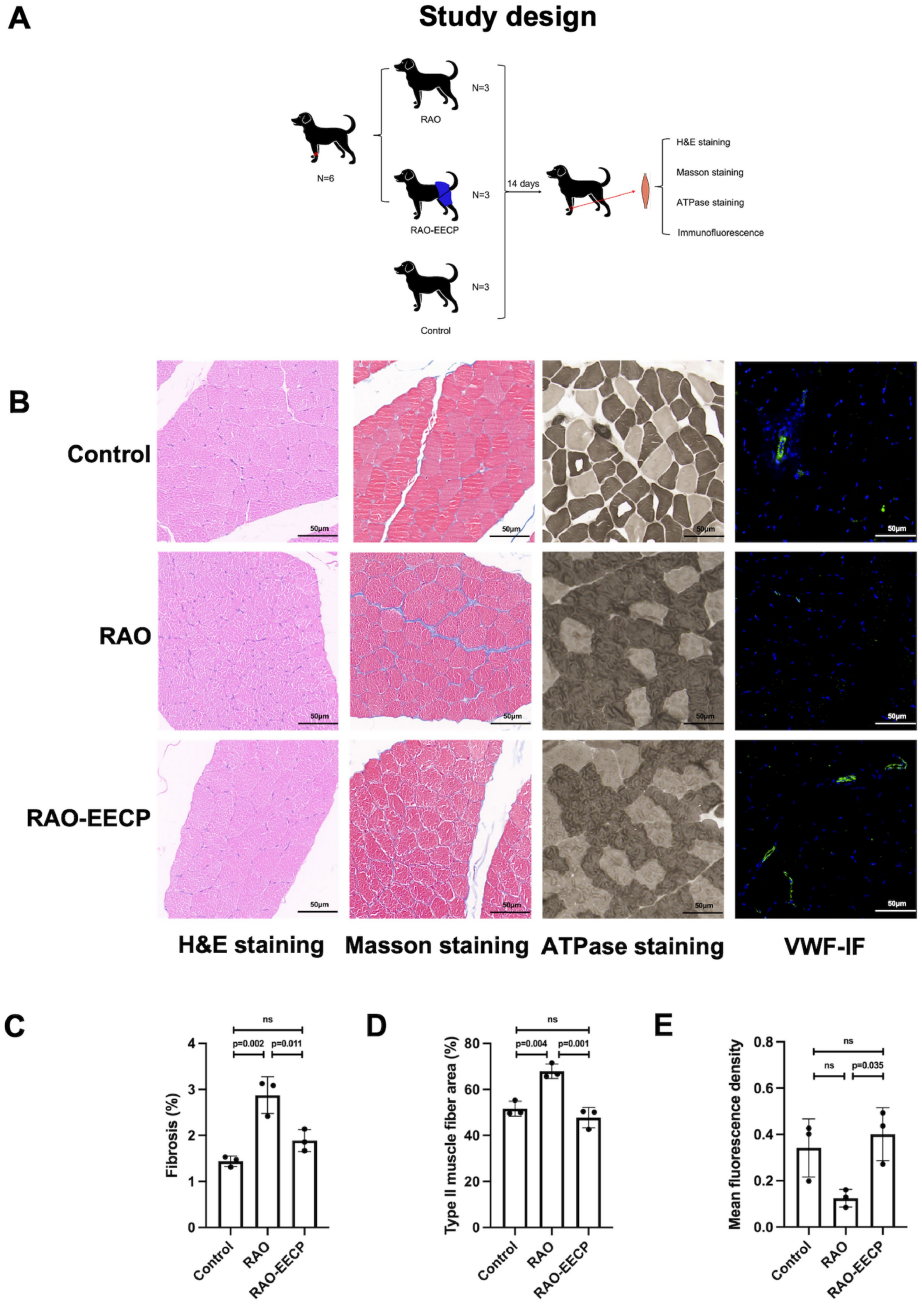
EECP promotes angiogenesis, improves muscle damage and aerobic metabolism. **(A)** Animal study design. **(B)** From left to right: H&E staining, Masson staining, ATPase staining and VWF immunofluorescence (IF) of cross section of lateral muscle of left forearm of Labrador dogs in three groups. Scale bars, 50 μ m. There was no significant difference in H&E staining among the three groups. VWF, Von Willebrand Factor. **(C)** Quantification of fibrotic regions (appearing as blue in Masson’s trichome in **C**). Data were expressed as means ± SD (*n* = 3 per group). Results were evaluated by one-way ANOVA followed by Tukey HSD test. **(D)** Quantification of type II muscle fibers (appearing as dark grey in **B**). Data were expressed as means ± SD (*n* = 3 per group). Results were evaluated by one-way ANOVA followed by Tukey HSD test. **(E)** Quantification of VWF immunofluorescence. Data were expressed as means ± SD (*n* = 3 per group). Results were evaluated by one-way ANOVA followed by Tukey HSD test.

In accordance with the predetermined research protocol (Figure 4A), we completed the establishment of the Labrador dog’s RAO model, the EECP treatment, the collection of skeletal muscle samples and related tests. The pathological staining results showed that (Figure 4B), there was no significant difference in the Hematoxylin eosin staining of the lateral muscle of left forearm among the three groups. However, Masson staining showed that the interstitial fibrosis of lateral muscle of left forearm caused by RAO (*p* = 0.002, 95%CI, 0.748–2.128), while after the treatment of EECP, the interstitial fibrosis was significantly reduced (*p* = 0.011, 95%CI, −1.676 to −0.296, Figure 4C). ATPase staining showed that type II muscle fibers in RAO group were significantly increased compared with control group (*p* = 0.004, 95%CI, 7.181–25.326), while type II muscle fibers were significantly decreased after treatment with EECP (*p* = 0.001, 95%CI, −29.213 to −11.069, Figure 4D). Immunofluorescence showed that the expression of VWF was significantly up-regulated in lateral muscle of left forearm after treatment with EECP (*p* = 0.035, 95%CI, 0.024–0.528, Figure 4E), which proved that EECP increased angiogenesis.

## Discussion

The real-world reported incidence of RAO remains the most frequent postprocedural complication of transradial access, with limited choice in the uptake of RAO therapeutic strategies. The reduction of sheath and catheter size (18), use of intraprocedural heparin (14), and maintenance of radial artery patency during hemostasis and oral anticoagulation (16) after TRA have been shown to lower the risk of RAO and have been termed best practices. Recently, The PROPHET-II Randomized Trial showed that prophylactic ipsilateral ulnar artery compression during radial artery hemostasis could significantly reduce the risk of RAO at the time of 24 h and 30 days after the procedure (73). However, there is no dedicated devices capable of dual compression on the market at present, which poses a great challenge for the wide application. In addition, recanalization of the occluded radial artery *via* the distal radial access (DRA) was reproted to be safe and effective, but the available evidence remains currently limited (74).

OS will be produced at the artery stenosis site, which further will lead to vascular dysfunction and atherosclerosis (21). In this study, we used the OS and PS models of HUVECs to analyze the longitudinal time-series of HUVECs mRNA. We finally found that with the prolongation of OS intervention, cell cycle, and vascular endothelial cell proliferation and angiogenesis response were down-regulated. At the same time, the inflammatory and oxidative stress responses and the related pathways of anaerobic metabolism of HUVEC were up-regulated. In addition, we found that PS intervention can significantly up-regulate the muscle synthesis, angiogenesis and NO production related molecules of HUVECs relative to OS, and significantly down-regulate the Inflammation and oxidative stress related molecules. This phenomenon may explain why arterial stenosis is aggravated, and the importance of PS for maintaining vascular function.

Our previous studies showed that EECP intervention can produce characteristic double pulse blood flow, and endothelial protective laminar flow to coronary artery, so as to restore vascular endothelial function by increasing pulsating shear stress and changing the expression of shear stress response gene, slow down the progress of atherosclerosis by regulating the proinflammatory pathway, and promote coronary collateral and angiogenesis (13, 23–25). However, there is no relevant research to prove whether EECP can produce similar blood flow pattern and function to coronary artery for radial artery.

In this study, we first evaluated the hemodynamic changes of the radial artery under the intervention of EECP through invasive radial artery pressure monitoring. We also found that the most appropriate counterpulsation pressure was 30Kpa. According to Hagen Poiseuille and Navier Stokes formulas (75), blood viscosity and the diameter of the vascular cavity remain unchanged, and the pressure gradient is proportional to the shear stress. We prove that EECP can generate double pulse blood flow and significantly increase the blood flow shear stress.

After that, we artificially made the local OS of the radial artery through the RAO model of Labrador dogs, and observed the histological changes of the muscle around the radial artery. At the same time, we used the EECP protocol (30Kpa, 1 h/day) to treat other RAO dogs for 14 days. The results showed that H&E staining had no serious muscle damage in the three groups, but Masson staining showed that RAO might cause interstitial fibrosis in local muscles, indicating that RAO may cause certain inflammatory reaction to surrounding muscle tissue, and muscle interstitial fibrosis was significantly reduced after treatment with EECP. In addition, ATPase staining demonstrated that RAO may cause a significant increase of type II muscle fibers in local muscle tissue, while type II muscle fibers are significantly reduced and type I muscle fibers are significantly increased after treatment with EECP. Researches showed that type II muscle fibers are mainly responsible for anaerobic metabolism, while type I muscle fibers are mainly responsible for aerobic metabolism (76). Therefore, we judged that RAO may cause insufficient local perfusion, leading to ischemia and hypoxia of muscle tissue, and EECP may improve this pathological state. Finally, we evaluated angiogenesis through the vascular marker VWF. The IF results showed that EECP may significantly increase angiogenesis in the local muscle tissue of the radial artery.

### Limitations and future research

Our research also has some limitations. There are some differences between the potential causes of RAO in clinical patients and the RAO animal model in this study, including the atherosclerosis, vascular access, artistic cather, exposure to wires, etc. Therefore, the results of this study cannot fully reflect the clinical efficacy of EECP on RAO. In addition, we have only verified in animal tests that the pulsating shear stress generated by EECP, and have not further performed clinical verification on patients with RAO stenosis. We will further conduct a multicenter randomized controlled trial to further verify the benefits of EECP for RAO patients.

## Conclusion

EECP can produce characteristic double pulse blood flow shear stress, and the radial artery peak pressure reaches the highest under the action of 30Kpa counterpulsation pressure. In addition, EECP may improve RAO induced interstitial fibrosis and hypoxia of muscle tissue around the radial artery, and increase angiogenesis of muscle tissue around the radial artery.

## Data availability statement

The datasets presented in this study can be found in online repositories. The names of the repository/repositories and accession number(s) can be found in the article/Supplementary material.

## Ethics statement

The animal study was reviewed and approved by Medical Research Ethics Committee of the Eighth Affiliated Hospital of Sun Yat-sen University (Futian, Shenzhen) and affiliated to the Eighth Affiliated Hospital of Sun Yat-sen University (Futian, Shenzhen).

## Author contributions

ZW and GW proposed the scientific problems. ZW and CY designed the experiments. ZW and JZ processed bioinformatics analysis. ZW, JL, XZ, JS, and WW processed and collected the animal experimental data. ZW and CY processed and calculated. All authors contributed to the article and approved the submitted version.

## Funding

This work was supported by the National Key R&D Program of China [2020YFC2004400]; National Natural Science Foundation of China [82270477 and 81970367]; Shenzhen Key Medical Discipline Construction Fund (no. SZXK002); Shenzhen Key Clinical Discipline Funds (ZDXKJF-01002); Shenzhen Science and Technology Innovation Committee [JCYJ20160608142215491]. National Natural Science Foundation of China [grant no. 82202292]. Guangdong Medical Science and Technology Research Foundation (no. A2022383) and Guangdong Basic and Applied Basic Research Foundation (no. 2021A1515110738). Shenzhen Futian District Health System Research Foundation (FTWS2022037). Shenzhen Futian District Health System Research Foundation (FTWS2020009).

## Conflict of interest

The authors declare that the research was conducted in the absence of any commercial or financial relationships that could be construed as a potential conflict of interest.

## Publisher’s note

All claims expressed in this article are solely those of the authors and do not necessarily represent those of their affiliated organizations, or those of the publisher, the editors and the reviewers. Any product that may be evaluated in this article, or claim that may be made by its manufacturer, is not guaranteed or endorsed by the publisher.
