# Supplementary material for: Enhanced external counterpulsation improves dysfunction of forearm muscle caused by radial artery occlusion
Source: Front Cardiovasc Med. 2023 Mar 2;10:1115494. doi: 10.3389/fcvm.2023.1115494 (PMC10022471; doi:10.3389/fcvm.2023.1115494)
Supplement: Supplementary file 4 [file Data_Sheet_1.docx]

Supplementary Methods

Enhanced External Counterpulsation Improves Dysfunction of Forearm Muscle Caused by Radial Artery Occlusion

**MORPHOLOGICAL EVALUATION**

**Hematoxylin-Eosin staining**

The experimental protocol for this part of study is as follows: Cut the paraffin embedded skeletal muscle sample into a 4μm thick cross section and mount it in 45 ℃ distilled water. After that, the slides are put into 60 ℃ for drying, dewaxed with xylene, and then soaked in absolute ethanol, 95% ethanol, 90% ethanol, 80% ethanol, and 70% ethanol for 5 minutes respectively for gradient xylene removal. Soak and clean with distilled water for 5 minutes, fully dye the hematoxylin dye solution for 10 minutes, and clean the excess hematoxylin dye solution with flowing water, Then use 1% hydrochloric acid ethanol (concentrated hydrochloric acid: absolute ethanol=1:100) for color separation for 10 seconds, then use flowing water to continuously clean the blueness for 15 minutes, rinse the glass slide with distilled water, and finally use light microscope to check the color separation quality of the nucleus. After confirming that the nuclear quality is satisfactory, use eosin dye solution to fully dye the above samples for 5 minutes. After rinsing with distilled water, successively use 70% ethanol, 80% ethanol, 90% ethanol, 95% ethanol and anhydrous ethanol to soak for 5 minutes for dehydration. Soak the dehydrated tissue sample slices with xylene for 5 minutes, then dry the tissue sample slices. Finally, use neutral gum seal, observe and take photos with a light microscope. The CaseViewer 2.4.0 automatic image analysis system was used to measure the parameters at 40 times. In order to evaluate the differences of the damaged muscle fibers among the three groups of dogs, Image J 1.53a software was used to conduct quantitative analysis of the damaged muscle fibers of the three groups of dogs. For each skeletal muscle sample, at least three different sections were analyzed.

**Masson staining**

The experimental protocol for this part of study is as follows: Cut the paraffin embedded skeletal muscle sample into a 4μm thick cross section and mount it in 45 ℃ distilled water, then place the glass slide in 60 ℃ for drying, dewax it with xylene, successively immerse it in anhydrous ethanol, 95% ethanol, 90% ethanol, 80% ethanol and 70% ethanol for 5 minutes respectively for gradient xylene removal, soak it in distilled water for 5 minutes, and put the glass slide in 2.5% potassium dichromate mordant solution for about 15 hours at room temperature, Immerse the slides in 2.5% potassium dichromate mordant solution and incubate them at 65 ° for 30 minutes. Wash them with flowing water for 30 seconds until the yellow color on the tissues fades. At the same time, preheat the ponceau acid fuchsin solution and 2.5% aniline blue solution to 65 ℃. Immerse the slides in the currently configured Weigert iron hematoxylin dye solution for 1 minute and then wash them with flowing water. Use 1% hydrochloric acid ethanol (concentrated hydrochloric acid: anhydrous ethanol=1:100) to differentiate for 1 minute until the nucleus is grayish black, and the background is almost colorless or light gray. Use flowing water to wash and distilled water to rinse successively, then immerse in ponceau acid fuchsin solution for 6 minutes, use distilled water to rinse, and then immerse in 1% phosphomolybdic acid solution for about 1 minute to differentiate collagen fibers, and then directly put the slide into 2.5% aniline blue solution for 30 seconds, Use three cylinders of 1% glacial acetic acid to rinse the differentiation glass slide for three times, each time for about 10 seconds, then use three cylinders of anhydrous ethanol to dehydrate for about 5 seconds, 10 seconds, 30 seconds, use two cylinders of n-butanol to dehydrate for 30 seconds and 2 minutes, then use two cylinders of xylene transparent, each time for 5 minutes, finally use neutral gum seal, use light microscope to observe and take photos. The CaseViewer 2.4.0 automatic image analysis system was used to measure the parameters at 40 times. In order to evaluate the differences of collagen fibers in the three groups of dog skeletal muscle samples, the Image J 1.53a software was used to conduct quantitative analysis of the collagen fibers in the three groups of dog skeletal muscle samples. For each skeletal muscle sample, at least three different sections were analyzed.

**ATPase staining**

Anaerobic metabolism of type II muscle fibers was evaluated by ATPase staining. The experimental protocol for this part of study is as follows: Prepare ATPase stain: it contains glycine sodium chloride stock solution (0.75g glycine, 0.58g sodium chloride, and 100ml distilled water), 1mol/L calcium chloride stock solution (14.7g CaCl2 • 2H2O, and 100ml distilled water), 0.1mol/L sodium hydroxide stock solution (10ml 1mol/L sodium hydroxide, and 90ml distilled water). ATPase working solution: 50ml of glycine sodium chloride, 10ml of 1mol/L CaCl2 • 2H2O, 35ml of 0.1mol/L sodium hydroxide stock solution. Use 1mol/L sodium hydroxide to adjust the working solution pH to 9.5-9.6. ATPase culture solution: ready to use, including 10ml ATPase working solution, 5mg adenosine triphosphate disodium for injection, pH 9.4. 1% calcium chloride solution: CaCl2 • 2H2O 2g, add distilled water to 200ml. 2% cobalt chloride solution: 4g of cobalt dichloride, and 200ml of distilled water. Staining: cut the skeletal muscle sample embedded in OCT into a 4μmthick cross section and dry it in the air, incubate it in 37 ℃ ATPase culture solution for 1h at 37 ℃, then put it into 1% calcium chloride solution and shake it in a shaker at room temperature for 5 minutes. Then put the sample into 2% cobalt chloride solution and shake it for 5 minutes with a shaker at room temperature. Put the sample in distilled water and use a shaker to clean the sample for 5 minutes, and repeat the cleaning for 3 times. Immerse the sample in ammonium sulfide diluent (ammonium sulfide 500ul, 10ml distilled water) for about 30 seconds until black appears, then use flowing water to wash for 10min, finally use 95% and absolute ethanol to dehydrate, make xylene transparent and fix it with neutral gum. Use CaseViewer 2.4.0 automatic image analysis system to measure parameters at 40 times. In order to compare the differences between type I and type II muscle fibers in the three groups of dogs, image J 1.53a software was used to quantitatively analyze the ATP enzyme staining sections of the three groups of dogs. For each skeletal muscle sample, at least three different sections were analyzed.

**Immunofluorescence protocol**

Put the paraffin section into the environment-friendly dewaxing solution, absolute ethanol and distilled water to dewax to water in turn, and then put the tissue section into a repair box filled with EDTA antigen repair buffer (PH8.0) for antigen repair in the microwave oven. After the section is slightly dried, draw a circle around the tissue with a histochemical pen (to prevent the antibody from flowing away), drop BSA in the circle and incubate it for 30 minutes, and then gently shake off the sealing solution, Add Von Willebrand Factor VWF first antibody (abcam6994, 1:1000) on the slice, and incubate the slice in a wet box at 4 ° C overnight. (Add a small amount of water in the wet box to prevent the antibody from evaporating). Put the glass slide in PBS (PH7.4), shake and wash it on the decolorization shaker for 3 times, 5min each time. After the sections are slightly dried, the corresponding secondary antibody covering tissue is dropped into the circle and incubated at room temperature for 50 min. After the slices are slightly dried, DAPI dye solution is dripped into the ring and incubated at room temperature in dark for 10 minutes. After the sections are dried, the spontaneous fluorescence quenching agent is added into the ring for 5min, and the water is washed for 10min. Put the glass slide in PBS (PH7.4), shake and wash it on the decolorization shaker for 3 times, 5min each time. Slightly dry the slices and then seal them with anti-fluorescence quenching sealing agent. Finally, the sections were photographed under the fluorescence microscope.
